# Effects of Nutritionally Induced Obesity on Metabolic Pathways of Zebrafish

**DOI:** 10.3390/ijms24031850

**Published:** 2023-01-17

**Authors:** Xixi Li, Guodong Ge, Guili Song, Qing Li, Zongbin Cui

**Affiliations:** 1 Guangdong Provincial Key Laboratory of Microbial Culture Collection and Application, State Key Laboratory of Applied Microbiology Southern China, Institute of Microbiology, Guangdong Academy of Sciences, Guangzhou 510070, China; 2 State Key Laboratory of Freshwater Ecology and Biotechnology, Institute of Hydrobiology, Chinese Academy of Sciences, Wuhan 430072, China

**Keywords:** Zebrafish, obesity, intestinal microflora, transcriptome sequencing, signal pathways

## Abstract

Human obesity has become a global epidemic that can lead to many metabolic diseases, including insulin resistance, type 2 diabetes, dyslipidemia, hypertension and nonalcoholic fatty liver. The development of obesity is closely associated with excess food intake and energy imbalance, family history, lifestyle, psychology and other factors, but molecular mechanisms underlying the induction and development of obesity remain to be intensively studied under a variety of internal and external pathogenesis conditions. In this study, we generated two obesity models of zebrafish that were treated with a high-fat diet (HFD) or an overfeeding diet (DIO). Both HFD and DIO zebrafish exhibited higher levels of lipid accumulation, fat distribution, microvascular steatosis and ectopic accumulation of lipid droplets in liver and muscle than normal diet (NOD) fish. The comparison of transcriptome sequencing data for the livers of HFD, DIO and NOD groups identified common and specific genes and signaling pathways that are potentially associated with zebrafish obesity induced by HFD and/or DIO. These findings provide clues for further understanding the mechanisms of obesity development and preventing nutritionally induced obesity through targeting the common signaling pathways and biological processes.

## 1. Introduction

The prevalence of obesity has increased worldwide in recent years, and the incidence of obesity is continuing to rise at an alarming rate. The World Health Organization (WHO) defines obesity as an excessive fat accumulation that might impair human health and having a body mass index (BMI) greater than 30 kg/m^2^ [1]. Moreover, obesity represents a major health challenge because it substantially increases the risk of diseases such as type 2 diabetes mellitus, fatty liver disease, hypertension, myocardial infarction, stroke, dementia, osteoarthritis, obstructive sleep apnoea and several cancers, thereby contributing to a decline in both quality of life and life expectancy [2]. The major drivers of the obesity epidemic are long-term energy imbalance between too many calories consumed and too few calories expended [3]. However, the pathogenesis of obesity has been proven to be more complex [4].

The development of obesity is closely associated with genetic predisposition, environmental and behavioral factors that can lead to increased physical inactivity and calorie intake [5,6]. Previous studies have attempted to characterize the whole-animal responses to high-calorie and high-fat diets and generally shown that the obesity phenotype can differ in the dietary protocols [5,7,8,9], suggesting the importance of developing and analyzing diet-induced models of obesity. 

The liver is the central organ that controls lipid homeostasis by means of complex but precisely regulated biochemical, signaling and cellular pathways [10], and it plays a fundamental role in coordinating systemic metabolic homeostasis and the adaptation to nutrient availability and deprivation [11]. Since the liver is the major organ of lipogenesis and lipid oxidation, the impaired hepatic lipid metabolism in the liver is tightly correlated with obesity, diabetes, and fatty liver disease [12].

Zebrafish is an attractive model animal for studying metabolic diseases because of the functional conservation in lipid metabolism, adipose biology, pancreas structure, and glucose homeostasis, and it is also suitable for the identification of novel targets associated with the risk and treatment of obesity and diabetes in humans [13]. Zebrafish has the key organs that are important for the regulation of energy homeostasis and metabolism in mammals, including digestive organs, adipose tissues, and skeletal muscle [14]. The key functions, such as appetite regulation, insulin regulation and lipid storage, are also well conserved in zebrafish [15,16,17]. Models of obesity and transgenic reporter lines for adipose tissue would be an excellent resource for high-throughput screening of potential drug targets for the treatment of obesity [18].

In this study, we generated two obesity models of zebrafish using high-fat diet (HFD) and overfeeding diet (DIO), respectively. The liver transcriptomic sequencing and comparison analysis of physiological normal diet (NOD), DIO and HFD groups were performed to identify key genes and signaling pathways that are potentially associated with HFD- and DIO-induced obesity in zebrafish.

## 2. Results

### 2.1. Obese Phenotypes Induced by HFD and DIO in Zebrafish

**Figure 1:**
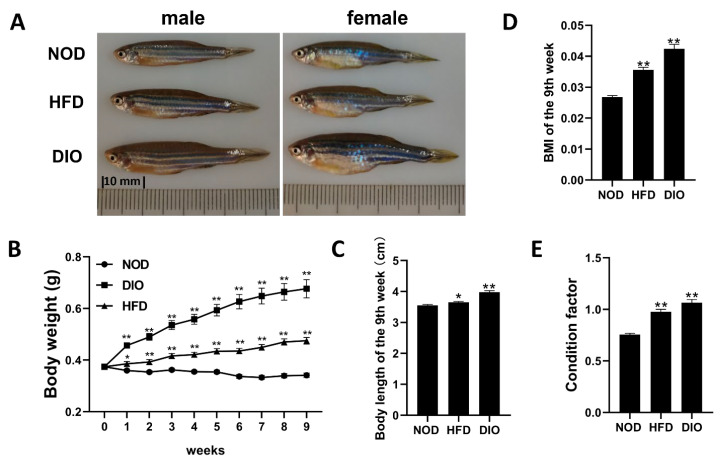
Both HFD and DIO induced obese phenotypes in zebrafish. (**A**) Lateral views of NOD, DIO and HFD zebrafish after 9 weeks of feeding. The distance between two dashes is 10 mm. (**B**) Changes in body weight during 9-week feeding experiments. (**C**) Changes in body length after 9 weeks of feeding. (**D**) Changes in BMI after 9 weeks of feeding. (**E**) Changes in condition factor after 9 weeks of feeding. *, *p* < 0.05, **, *p* < 0.01. Values are means ± SEM. Each group has 60 fish.

After feeding with NOD, DIO or HFD for 9 weeks, we assessed the nutritional obesity of adult zebrafish. Male and female zebrafish in both DIO and HFD groups showed an enlarged belly when compared to those in the NOD group (Figure 1A). The average body weights of DIO and HFD groups were 1.98 and 1.39-fold higher (*p* < 0.01) than those of the NOD group, respectively (Figure 1B). The weight gains observed in DIO and HFD groups were accompanied with a significant increase in body length (Figure 1C). Moreover, the body mass index (BMI, Figure 1D) and condition factor (CF, Figure 1E) of DIO and HFD groups were significantly higher than those of the NOD group. These results indicate that rearing zebrafish with DIO or HFD successfully induced an obese phenotype.

### 2.2. Both HFD and DIO Induced Excess Fat Distribution and Lipid Accumulation in Zebrafish

**Figure 2:**
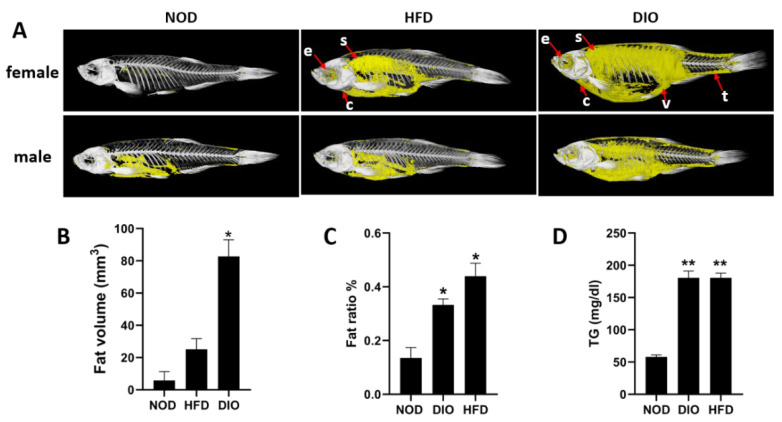
Lipid accumulation and fat distribution in three groups. (**A**) The in vivo micro-CT scans of zebrafish. Each group measures 4 fish, including 2 females and 2 males. Yellow: adipose tissue; white: bone; e: eyes; s: subcutaneous; c: heart; v: viscera; t: tail. (**B**) Quantitative analysis of the fat volume. Each group measures 4 fish, including 2 females and 2 males. Values are means ± SEM. (**C**) Total lipid content of whole fish. (**D**) Plasma TG levels. *, *p* < 0.05, **, *p* < 0.01. Values are means ± SEM. n = 4 for each group.

To quantify the fat volume in the total body of zebrafish, assays using in vivo micro-CT (micro-computed tomography) scans were performed. As shown in Figure 2A, there was only a small amount of fat accumulation in the viscera of NOD zebrafish, but a large amount of fat accumulation in the abdomen, subcutaneous, eyes, and around the heart and tail of both HFD and DIO zebrafish. Total body fat volumes in the DIO and HFD groups were significantly greater than those in the NOD group (Figure 2B). Consistently, the total lipid content of whole fish was significantly increased in both HFD and DIO fish (Figure 2C). Plasma triglyceride (TG) levels were also significantly (*p* < 0.01) higher in the HFD and DIO groups than in the NOD group after feeding for 9 weeks (Figure 2D). Thus, HFD and DIO zebrafish exhibited a significantly higher degree of fat distribution and lipid accumulation than NOD zebrafish.

### 2.3. HFD and DIO Caused Hepatic Steatosis and Ectopic Accumulation of Lipid Droplets in Liver and Muscle

**Figure 3:**
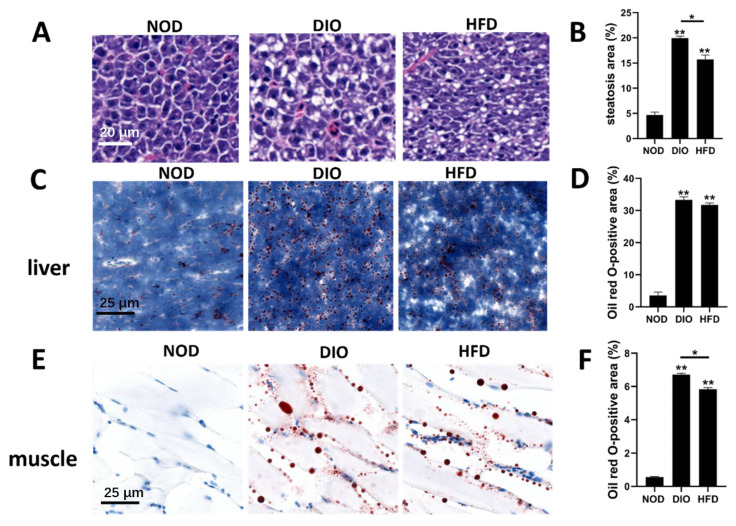
Characterization of hepatic steatosis and ectopic accumulation of lipid droplets in the liver and muscle. (**A**) Livers of zebrafish in NOD, DIO and HFD groups were analyzed with hematoxylin and eosin staining. (**B**) Quantitative analysis of the area of hepatic steatosis in zebrafish. Steatosis of three NOD, three HFD and three DIO across the liver were determined using the ImageJ software. Values are means ± SEM. **, *p* < 0.01. (**C**,**E**) Livers and muscles of zebrafish in NOD, DIO and HFD groups were analyzed with Oil Red O. (**D**,**F**) Quantitative analysis of the area of lipid droplets in liver and muscle. The areas of lipid droplets in the liver and muscle of three NOD, three HFD and three DIO were determined using the ImageJ software. Values are means ± SEM. *, *p* < 0.05. **, *p* < 0.01.

To characterize the effects of NOD, HFD and DIO on tissue structure, liver and muscle were collected and subjected to histological analyses. After 9 weeks of feeding, the livers of three groups were analyzed with hematoxylin and eosin staining. As shown in Figure 3A, a severe steatosis was observed in the livers of the DIO and HFD groups. The areas of hepatic steatosis in DIO and HFD groups were significantly larger than in the NOD group (Figure 3B). To evaluate whether lipids accumulated in the liver and muscle, hepatic and muscular tissues were stained with Oil Red O. A prominent ectopic accumulation of lipids in liver and muscle was observed in HFD and DIO groups (Figure 3C,E). The areas of lipid droplets in the liver and muscle in the DIO and HFD groups were significantly larger than in the NOD group (Figure 3D,F). Together, these results indicate that HFD and DIO caused hepatic steatosis and ectopic accumulation of lipid droplets in liver and muscle.

### 2.4. Identification of Differentially Expressed Genes (DEGs) Induced by HFD and DIO in Zebrafish

**Figure 4:**
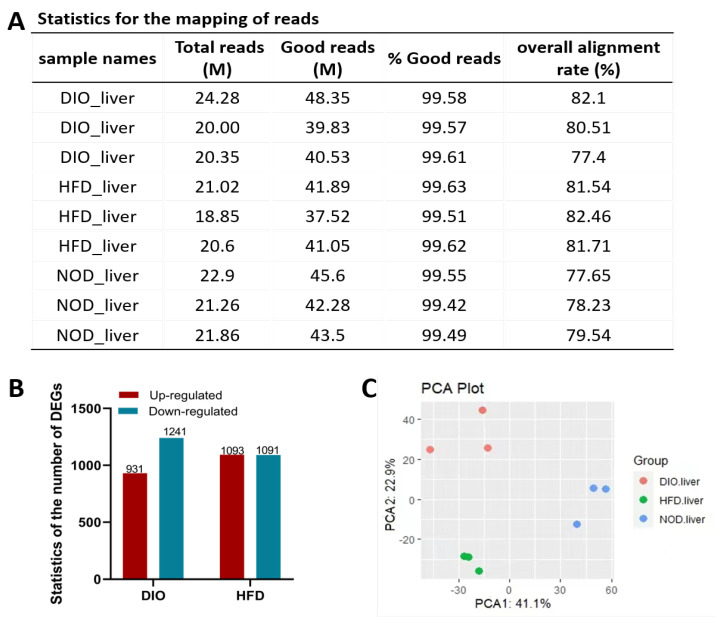
Identification of DEGs induced by HFD and DIO in zebrafish. (**A**) Statistics for the mapping of reads in three groups. (**B**) The number of DEGs between groups of different diets (fold change ≥ 1.5 and *p*-value ≤ 0.05). (**C**) The principal component analysis (PCA) of differentially expressed genes in three groups.

To understand signaling pathways potentially controlling the induction of obesity by HFD and/or DIO in zebrafish, nine cDNA libraries of livers from zebrafish in the NOD, DIO and HFD groups were constructed and subjected to high-throughput RNA-seq, followed by bioinformatics analysis. RNA-seq analysis generated 18.85–24.28 million (M) pairs of raw reads for each of the samples, and about 77.4–82.46% of the processed reads were mapped to the reference genome of zebrafish (Figure 4A). 

The comparison of RNA-seq data produced two groups of DEGs with a fold change of ≥ 1.5 and a *p*-value ≤ 0.05, and the details of these genes are listed in Table S1. There are 931 up-regulated genes and 1241 down-regulated genes in group DIO, and 1093 up-regulated genes and 1091 down-regulated genes in group HFD (Figure 4B). The first (PC1) and second (PC2) principal component analyses (PCA) of differentially expressed genes in three groups showed variations of 41.1% and 22.9%, indicating a clear separation of genes in different groups (Figure 4C).

### 2.5. GO Enrichment of DEGs Induced by HFD and DIO in Zebrafish 

**Figure 5:**
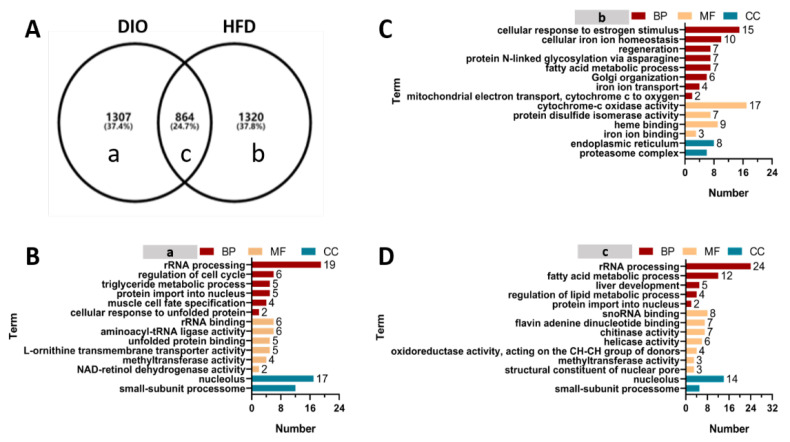
GO enrichment analysis of DEGs in (a), (b) and (c) groups. (**A**) Venn diagram analysis of differentially expressed genes. a–c: different letters represent genes specifically expressed in different Venn groups. (**B**) GO enrichment analysis of genes specifically expressed in group (a) that represent a class of genes specifically expressed in DIO zebrafish. (**C**) GO enrichment analysis of genes specifically expressed in group (b) that represent a class of genes specifically expressed in HFD zebrafish. (**D**) GO enrichment analysis of genes specifically expressed in group (c) that represent a class of genes shared in DIO and HFD zebrafish.

To further explore the differences in biological processes (BP), cellular composition (CC) and molecular functions (MF) in DIO and HFD, all DEGs were divided into three groups by a Venn diagram analysis (a–c) (Figure 5A; Table S2). The DEGs in group (a) represent genes specifically expressed in the DIO group, which account for 37.4% of all DEGs. The DEGs in group (b) were specifically expressed in the HFD group, which account for 37.8% of the total DEGs. The DEGs in group (c) were shared by both the DIO and HFD groups, which account for 24.7% of all DEGs. 

Then, GO enrichment analysis of the DEGs in groups (a), (b) and (c) were performed. Totals of 185 GO terms for (a), 171 GO terms for (b) and 180 GO terms for (c) were enriched based on the *p*-value ≤ 0.05 and count ≥ 2 (Table S3). Representatives of the GO term through the REVIGO tool are displayed in Table S4. GO terms were clustered into three hierarchies, including biological process (BP), molecular function (MF) and cellular component (CC). 

The DEGs in group (a) were primarily enriched in rRNA processing, regulation of cell cycle, triglyceride metabolic process and protein import into nucleus in BP, rRNA binding, aminoacyl-tRNA ligase activity, unfolded protein binding and L-ornithine transmembrane transporter activity in MF, and nucleolus and small-subunit processome in CC (Figure 5B).

The most overrepresented GO terms for DEGs in group (b) were cellular response to estrogen stimulus, cellular iron ion homeostasis, regeneration, protein N-linked glycosylation via asparagine and fatty acid metabolic process in BP, cytochrome-c oxidase activity, protein disulfide isomerase activity and heme binding in MF, and endoplasmic reticulum and proteasome complex in CC (Figure 5C). 

The DEGs in group (c) overlapped in both DIO and FFD groups, which were primarily enriched in rRNA processing, fatty acid metabolic process, regulation of lipid metabolic process and liver development in BP, snoRNA binding, flavin adenine dinucleotide binding, chitinase activity, helicase activity and oxidoreductase activity in MF, nucleolus and small-subunit processome in CC (Figure 5D). 

### 2.6. KEGG Enrichment of Signaling Pathways Induced by HFD and DIO in Zebrafish 

KEGG enrichment analysis was performed to reveal the functional characteristics of DEGs in three groups, respectively (Table S5). Since one gene is usually mapped to different signaling pathways, the Jaccard coefficient was introduced to calculate the distance between two signaling pathways according to the proportion of genes they shared. The networks of KEGG pathways in three groups were obtained by the Jaccard coefficient (Table S6), and CytoHubba was used to identify the hub pathways in the networks. 

**Figure 6:**
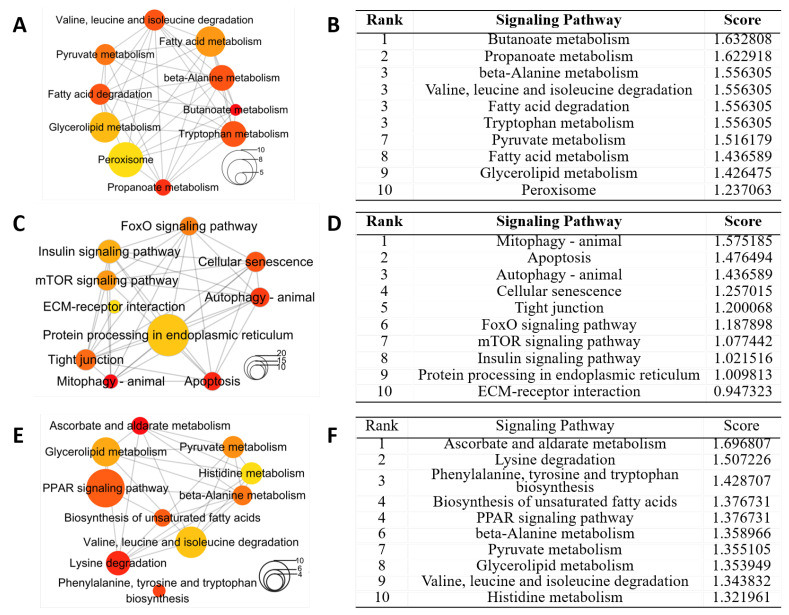
Hub signaling pathways from KEGG enrichment analysis of DEGs in livers for different groups. Networks of top 10 hub pathways (**A**) and top 10 hub pathways ranked by DMNC method (**B**) for DEGs in group (a). Networks of top 10 hub pathways (**C**) and top 10 hub pathways ranked by DMNC method (**D**) for DEGs in group (b). Networks of top10 hub pathways (**E**) and top 10 hub pathways ranked by DMNC method (**F**) for DEGs in group (c). Node color and size stand for the enrichment *p*-value and the number of genes in the pathway, respectively.

Among the signaling pathways enriched from the DEGs in group (a), the top three hub signaling pathways were butanoate metabolism, propanoate metabolism and beta-alanine metabolism (Figure 6A,B). Among the signaling pathways enriched from the DEGs in group (b), the top three hub signaling pathways were mitophagy—animal, apoptosis and autophagy—animal (Figure 6C,D). Among the signaling pathways enriched from DEGs in groups (c), the top three hub signaling pathways were ascorbate and aldarate metabolism, lysine degradation and phenylalanine, tyrosine and tryptophan biosynthesis (Figure 6E,F).

### 2.7. Enrichment of Hub Genes Associated with the Obesity Induced by HFD and/or DIO in Zebrafish 

**Figure 7:**
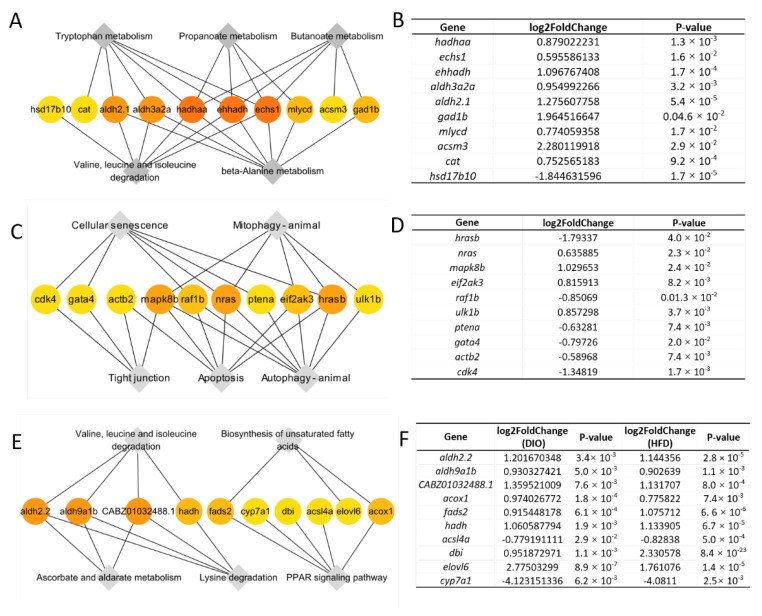
Hub genes within KEGG pathways. (**A**) Networks of 10 hub genes mapped to top 5 pathways in group (a). (**B**) The fold changes of hub genes in group (a). (**C**) Networks of 10 hub genes mapped to top 5 pathways in group (b). (**D**) The fold changes of hub genes in group (b). (**E**) Networks of 10 hub genes mapped to top 5 pathways in group (c). (**F**) The fold changes of hub genes in group (c).

We also examined the hub genes with CytoHubba. In group (a), the hub genes (*hadhaa, echs1, ehhadh, aldh3a2a, aldh2.1, gad1b, mlycd, acsm3, cat* and *hsd17b10*) were clustered into beta-alanine metabolism, tryptophan metabolism, valine, leucine and isoleucine degradation, propanoate metabolism and butanoate metabolism (Figure 7A,B). The hub genes (*hrasb, nras, mapk8b, eif2ak3, raf1b, ulk1b, ptena, gata4, actb2* and *cdk4*) in group (b) were clustered into tight junction, cellular senescence, autophagy—animal, apoptosis and mitophagy—animal (Figure 7C,D). Similar to those in groups (a) and (b), the hub genes (*aldh2.2, aldh9a1b, CABZ01032488.1, acox1, fads2, hadh, acsl4a, dbi, elovl6* and *cyp7a1*) in group (c) were clustered into PPAR signaling pathway, valine, leucine and isoleucine degradation, lysine degradation, biosynthesis of unsaturated fatty acids and ascorbate and aldarate metabolism (Figure 7E,F). 

Then we compared KEGG pathways enriched in groups (a), (b) and (c) (Tables S7 and S8). The beta-alanine metabolism was enriched in all of the three groups, but it is one of the hub pathways only found in group (a) (Figure 7A). Tryptophan metabolism and propanoate metabolism were enriched in groups (a) and (c), but they were hub pathways only in group (a) (Figure 7A). Valine, leucine and isoleucine degradation was one of the hub pathways found in groups (a) and (c) (Figure 7A,E). The PPAR signaling pathway was enriched in groups (b) and (c) (Figure 7C,E), but it was only a hub pathway in group (c) (Figure 7E). In addition, the KEGG pathways enriched in group (b) (Figure 7C) were not found in groups (a) and (c) (Figure 7A,E). These data indicate that the hub pathways in group (b) are unique for HDF.

## 3. Discussion

Obesity is a major global health problem caused by heredity and environmental factors, which is characterized by an increase in adipose tissue accumulation in the presence of positive energy balance. An increasing body of evidence indicates that obesity is a risk factor for metabolic and cardiovascular diseases, and premature mortality [19]. Zebrafish possess many structural and functional similarities to humans and have been used to study various human diseases, including obesity [9,18]. In this study, two obesity models of zebrafish were successfully induced by HFD or DIO. We showed that adult zebrafish fed with either DIO or HFD can show a significant increase in body weight, body length, BMI and CF in comparison with NOD zebrafish. In addition, HFD and DIO zebrafish present higher levels of fat distribution in many tissues, lipid accumulation in the whole fish, ectopic lipid droplets in liver and muscle, and hepatic steatosis. We further performed the transcriptional profiling of livers with RNA-seq to identify changes in hub pathways and key genes that are likely responsible for the obesity induced by DIO and/or HFD.

The Venn diagram analysis showed that 1307 DEGs were specifically detected in the DIO group, and 1320 DEGs in the HFD, indicating a big difference in effects between HFD and DIO in terms of gene expression. These findings were also noticed in a previous study showing that the short-term overfeeding of zebrafish with a normal-fat diet (NFD) or a high-fat diet (HFD) will develop metabolically healthy versus unhealthy obesity [9]. The study has also shown that 8 weeks overfeeding with either NFD or HFD can lead to a significant increase in body weight and AT mass when compared to controls. In contrast to NFD-overfed zebrafish, HFD-overfed zebrafish additionally present metabolic alterations, such as hyperglycemia and ectopic lipid accumulation in the liver, and a metabolically unhealthy AT phenotype with adipocyte hypertrophy especially in the visceral AT depot, which is accompanied by changes in the expressions of marker genes for lipid metabolism, inflammation and fibrosis.

In this study, we fond that zebrafish in both DIO and HFD groups have the obese phenotype, such as increased fat distribution and ectopic accumulation of lipid droplets in liver and muscle. The Venn diagram analysis showed that 864 DEGs were shared by DIO and HFD. The GO enrichment analysis showed that these 864 DEGs were enriched in lipid metabolic process, including fatty acid metabolic process, regulation of lipid metabolic process and liver development. The KEGG enrichment analysis also showed that these 864 DEGs were enriched in pathways associated with lipid metabolism, including glycerolipid metabolism, biosynthesis of unsaturated fatty acids and PPAR signaling pathway. These data indicate that lipid metabolism is the common pathway shared by obese zebrafish in both the DIO and HFD groups, which is consistent with other studies concerning obesity in zebrafish and mammals [6,9,20,21].

The Venn diagram analysis showed that 1307 DEGs were specifically detected in the DIO group. The KEGG enrichment analysis showed that these 1307 DEGs were enriched in pathways associated with short-chain fatty acids (SCFAs) metabolism, including butanoate metabolism, propanoate metabolism, pyruvate metabolism, fatty acid metabolism, glycerolipid metabolism and fatty acid degradation. These data indicate that the SCFAs metabolism is important to the obesity of zebrafish in the DIO group. SCFAs are important for host metabolism and are used as substrates for energy production, lipogenesis and cholesterol synthesis [22,23], and the aberrant production of SCFAs has emerged in obesity [24]. SCFAs can increase leptin secretion by activating FFAR2 in vivo or in vitro [25]. Leptin stimulates the oxidation of fatty acids [26] and the uptake of glucose [27,28], and prevents the accumulation of lipids [29]. Free fatty acids are taken up by hepatocytes and converted into triglycerides for storage with cholesterol in lipid droplets [30]. 

The Venn diagram analysis showed that 1320 DEGs were specifically detected in the HFD group. The GO enrichment analysis showed that the most representative GO term in the HFD group was cellular response to estrogen stimulus. In a transgenic mouse model designed to detect estrogen signaling, the liver was actually the most responsive to estrogen [31]. Estrogens stimulate serotonin neurons to inhibit binge-like eating in mice [32]. Estrogens synergize with adipose tissue genes to increase gluteofemoral subcutaneous adipose tissue mass and decrease central adipose tissue mass in reproductive-age women [33].

The KEGG enrichment analysis of the 1320 DEGs showed that the most representative KEGG pathway in the HFD group was mitophagy—animal. Mitophagy is an autophagic response that specifically targets damaged mitochondria [34] and plays an essential role in maintaining the health of the mitochondrial network [35,36]. Mitochondria are specialized organelles that act as metabolic hubs and signaling platforms, involved in an array of essential cellular processes such as ATP production and fatty acid oxidation [37,38]. These data indicate that mitochondria metabolism may play an important role in the obesity of zebrafish in the HFD group. 

The insulin signaling pathway was also enriched in the HFD group. Obesity is a strong risk factor for the development of type 2 diabetes mellitus [39]. Insulin-resistant individuals exhibit increased *de novo* lipogenesis and re-esterification, inducing fat accumulation in the liver [11]. In addition, insulin-resistant individuals have increased secretion and decreased clearance of triglyceride [40,41]. Ablation of the insulin-producing cells (IPCs) in the brain of *Drosophila* causes increased lipid stores [42,43]. These data indicate that the HFD model may be suitable for developing an understanding of insulin resistance.

In summary, the differntial effects of DIO and HFD on the liver metabolism of obese zebrafish provide clues for further understanding the mechanisms of obesity development and preventing nutritionally induced obesity by targeting the common signaling pathways and biological processes. 

## 4. Materials and Methods

### 4.1. Zebrafish Husbandry

AB strain was used in this study. All fish were maintained under standard laboratory conditions at 28 °C with a light/dark cycle of 14/10 h [44]. 

### 4.2. Zebrafish Feeding Experiments and Sampling

The feeding protocol used in this study was based on a published work [9]. The 6-month-old wild type (WT) zebrafish were randomly divided into three dietary groups: one group was fed with peeled Artemia salina cysts in a normal diet (NOD; 5 mg artemia per fish per day), another group was overfed with artemia to induce an obese state (DIO; 60 mg artemia), and a third group was fed a combination of artemia (5 mg artemia) and egg yolk powder (Sigma; 30 mg) mimicking a high-fat diet (HFD). Zebrafish were maintained at 20 fish per 10 L tank and fed three times per day. At week 9, zebrafish were fasted overnight and sacrificed. The 5 mg artemia per day corresponds to the physiological energy requirement of an adult zebrafish [14]. 

The body weight of zebrafish was measured weekly during the overfeeding treatment as previously described [45,46]. Briefly, fish were anesthetized with buffered tricaine. Tricaine was prepared at 0.02% concentration in facility water and the fish were transferred to a 10 cm dish containing the mixture. They were then monitored for the third stage of anesthesia in which there was loss of equilibrium, operculum movements and reactivity. Stage III was usually reached in a minute, after which measurements could be taken. Body weight (g) was measured after the body surface was dried with soft tissue paper (Ultra strong; Vinda; China). Fish were then allowed to recover from the anesthesia. 

The body weight and length of the anaesthetized zebrafish was measured weekly, and body mass index (BMI) and the condition factor (CF; CF = 100 × body weight/body length^3^) were calculated after the feeding treatment. 

### 4.3. Micro-CT

The body fat volume was measured as previously described [47]. Briefly, zebrafish were anesthetized using MS-222, restrained between two wet sponges. Whole zebrafish were scanned using a Micro-CT system (μCT-50; Scanco medical, Bassersdorf, Swizerland) at a resolution of 14 μm. The three-dimensional (3D) images of the adipose tissue were obtained by a 3D reconstruction with software VG Studio Max (v2.1). At least four adults in each group were scanned.

### 4.4. Total Lipid Measurement, Oil Red O Staining and HE Staining

Total lipid contents (percent dry weight) were measured using the Folch procedure as previously described [16]. The liver and muscle tissue were stained with Oil Red O and HE as previously described [48]. Microscopic images at 40x magnification were obtained. Image analyses were performed using ImageJ software.

### 4.5. Measurement of Plasma TG

The triglyceride (TG) contents were determined with commercial kits according to the manufacturer’s instructions (Nanjing Jiancheng Bioengineering Institute, Nanjing, China).

### 4.6. Sample Collection and RNA-seq Analysis

At week 9, zebrafish were fasted overnight and sacrificed. The livers and brains were collected and each group had three independent biological replicates. Thus, 18 sequencing libraries were constructed and sequenced. Library construction and high-throughput RNA-sequencing (RNA-seq) were performed by experts in the Analytical and Testing Center at the Institute of Hydrobiology, Chinese Academy of Sciences (http://www.ihb.ac.cn/fxcszx/, accessed on 29 November 2022). The methods for sample quality analysis and the preparation of the RNA library and RNA-seq were as previously described [49]. The bioinformatics analysis was conducted as previously described [50].

### 4.7. Statistical Analysis

The data are presented as mean ± standard deviation. Statistical differences between two sets of data were analyzed using two-tailed paired Student’s t-test, and a value of p < 0.05 was considered as significant.

## Data Availability

The data presented in this study are available in the article and supplementary material.

## Supplementary Materials

The supporting information can be downloaded at: https://www.mdpi.com/article/10.3390/ijms24031850/s1.
